# Anti‐*Trichuris* mucosal responses are maintained during *H. bakeri* co‐infection despite impaired parasite expulsion

**DOI:** 10.1111/pim.12936

**Published:** 2022-05-31

**Authors:** Stefano A. P. Colombo, Seona Thompson, Allison J. Bancroft, Richard K. Grencis

**Affiliations:** ^1^ Lydia Becker Institute of Immunology and Inflammation, Faculty of Biology, Medicine and Health Manchester Academic Health Science Centre, University of Manchester Manchester UK; ^2^ Wellcome Trust Centre for Cell Matrix Research, Faculty of Biology, Medicine and Health Manchester Academic Health Science Centre, University of Manchester Manchester UK

**Keywords:** cytokines, goblet cells, *Heligmosomoides bakeri*, *Heligmosomoides polygyrus*, intestinal mucosa, lymph nodes, Th2, Trichuris

## Abstract

In endemic regions concurrent infection with multiple gastrointestinal (GI) helminth species is more common than single species infection. However, the majority of model helminth infections focus on single species infections leading to a lack of understanding of how co‐infection influences anti‐parasite immune responses. Here, we use a model co‐infection of *Trichuris muris* (*Tm*) and *Heligmosomoides bakeri* (*Hb*) to investigate the effect of *Hb* on anti‐*Tm* immune responses. We observed a complete impairment of *Tm* expulsion in immune competent C57BL/6 mice when co‐infected with *Hb*. This was coupled with reduced cellularity in the colonic mesenteric lymph node (cMLN) proximal to the caecum, however, cMLN cytokine responses and caecal mucosal immune responses in co‐infected mice were not significantly different from mice infected with *Tm* alone. Interestingly, in immune‐compromised mice, we found co‐infection resulted in enhanced growth and fecundity of female *Tm* parasites. These data suggest that during helminth‐helminth co‐infection, immune‐independent signals between species may promote survival and growth.

## 1 INTRODUCTION

Infection with gastrointestinal (GI) helminths is one of the most common global infections of mammals.^1^ The consequences of such infections for global health and livestock production remain substantial.^2^, ^3^ A great deal of work has focused on understanding the immunological mechanisms that sway the balance between susceptibility and resistance to infection to individual GI helminth species.^4^, ^5^ However, isolating GI helminth species from one another, whilst a useful tool for reductionist experimentation, disregards the reality of infection *in natura* where concurrent infection with multiple GI helminths is the norm.^6^, ^7^, ^8^, ^9^ Whilst field data are limited, co‐infection in humans appears to have significant consequences for the phenotype and outcome of each infection. Individuals infected with multiple GI helminth species display higher burdens of each species relative to mono‐infected individuals, and tend to experience greater infection‐associated morbidity.^7^, ^9^ Despite the evident impact of co‐infection on health, it remains unclear whether increased infection burden and morbidity in co‐infected individuals is correlative (i.e., an individual susceptible to one species of GI helminth is simply more likely to also be susceptible to other species) or if there is a causative link (i.e., infection with one species renders you more susceptible to infection by other GI helminths).

There is limited data in experimental murine systems on how helminth‐helminth co‐infection impacts the kinetics and immune response of infection. In early work looking solely at infection burden it was observed that when *Heligmosomoides bakeri* (*Hb*, formerly *H. polygyrus* and *Nematospiroides dubius*) was co‐infected in models of resistance to *Trichuris muris* (*Tm*) or *Trichinella spiralis*, expulsion of these parasites was dramatically impaired.^10^, ^11^ These early data support the idea that infection with one GI helminth can induce susceptibility to chronic infection with other GI helminths in individuals which would have otherwise efficiently expelled those parasites. However, the mechanisms through which susceptibility to infection is conferred were not interrogated. More broadly, GI helminth co‐infection with pathogenic protozoan parasites, bacteria and viruses has been shown to impair immune responses to these co‐infecting species, and this is largely attributed to a skewing of the immune response from the required Th1 response to a more Th2‐dominated one,^12^, ^13^, ^14^, ^15^ although this may also be a consequence of the induction of regulatory/suppressor mechanisms. Additionally, helminth infection has been shown to reduce the efficacy of vaccination both in humans and in murine models.^16^, ^17^ Thus, there is strong evidence that infection with a helminth species can have significant consequences for immune responses to other stimuli, and it is conceivable that differential outcomes in helminth‐helminth co‐infections vs single‐species infections is due to influences exerted on the immune system.

*Tm* is a natural rodent whipworm closely related to the human‐infecting *Trichuris trichiura*.^18^, ^19^ Following ingestion of embryonated eggs, L1 larvae hatch and colonize the caecal epithelium. As they mature they extend their posterior end into the caecal lumen reaching into the proximal colon whilst their anterior end remains anchored within the epithelium. During chronic infection mating and egg production begins around 33 days post infection. Experimental high‐dose *Tm* infection (oral gavage of ≥200 embryonated eggs) has been used extensively as a model of resistance to GI helminth infection.^15^ C57BL/6 mice given a high‐dose of *Tm* develop a robust caecal/colonic IL‐13‐driven type‐2 immune response, characterized by increased epithelial cell turnover, goblet cell hyperplasia, and expression of type‐2‐associated mucosal genes, for example, *Muc5ac* and *Retnlb*.^20^, ^21^, ^22^, ^23^ These type 2 effector mechanisms efficiently expel *Tm* by 25–30 days post‐infection (prior to reaching patency). Activation of these mechanisms is dependent of Th2 derived IL‐13 signalling through IL‐4Rα2,^15^, ^24^, ^25^ however, the factors responsible for triggering Th2 polarization of naïve Th cells in this system are not well‐understood.

*Hb* is also a rodent‐specific enteric helminth which, unlike *Tm*, colonizes the proximal duodenum. Ingested L3 larvae rapidly penetrate through the duodenal epithelium, incubate in the muscularis externa/serosa, then erupt back into the intestinal lumen 8–10 days post infection as mature adults where they rapidly begin mating and releasing eggs.^26^ In C57BL/6 mice, despite inducing a potent type 2 immune, *Hb* persists as a relatively long‐lived chronic infection.^26^ Its persistence in the face of induction of anti‐helminth effector mechanisms has been associated with its ability to secrete immunomodulatory peptides, in particular TGFβ mimics and IL‐33 antagonists that potentially promote Treg cell expansion^27^ and dampen type 2 activation,^28^ to limit the immune response sufficiently to allow their survival. In the context of co‐infection with other helminths this creates interesting immunological possibilities, (i) the potent type 2 immune response induced could act to saturate the intestinal mucosa in type 2 signals enhancing resistance to other helminths which require type 2‐driven effector mechanisms, however, (ii) the secreted immunomodulatory peptides may instead render mice more susceptible to infection by dampening specific signals.

Here, we co‐infected C57BL/6 mice with *Tm* and *Hb* and assessed the immune response in the colonic mesenteric lymph node (cMLN) proximal to the caecum and caecal mucosa to assess how *Hb* infection impacts the immune response to *Tm*. We observed that impaired expulsion of *Tm* in co‐infected mice was associated with reduced cellularity particularly in the cMLN relative to mono‐infected mice. Interestingly, cytokine responses in the cMLN and physiological changes in the caecal mucosa were not significantly altered by co‐infection. We also demonstrate, through use of a model anti‐*Tm* immunization protocol, that co‐infection‐induced susceptibility does not suppress immunization‐induced immunity. Finally, we provide evidence that *Hb* co‐infection can enhance *Tm* growth and fecundity in an immune‐independent manner.

## 2 MATERIALS AND METHODS

### 2.1 Mice

Six‐week old C57BL/6 female mice were purchased from Envigo. Mice were housed in the University of Manchester Biological Services Facility for one week prior to the experimental start point. Female SCID mice were bred in‐house and were 7 weeks old at the experimental start point. All mice were housed under specific pathogen free conditions and the experiments were conducted in accordance with the Animals (Scientific Procedures) Act and following authorization by the local AWERB committee. All mice were euthanized by CO_2_ in rising concentration.

### 2.2 Parasite infections and immunizations

*Tm* eggs, *Hb* L3 larvae, and *Tm* excretory/secretory antigen (TmES) were collected from chronically infected mice as previously detailed.^29^ Mice were infected with *Tm* and/or *Hb* by oral gavage of *Tm* eggs and/or *Hb* L3 suspended in 200 μl of dH_2_O. For immunization with TmES, 50 μg of TmES was suspended in 200 μl of Alum using a magnetic stirrer and injected subcutaneously 21 days post *Hb* infection.

### 2.3 cMLN isolation, ex vivo stimulation, and cytometric bead array

At autopsy the cMLN was identified by anatomical position,^30^ isolated from the MLN chain, and placed into ice cold wash buffer (RPMI 1640 + 2% FCS + 100 U/ml penicillin + 100 μg/ml streptomycin, Gibco). cMLNs were homogenized by manually forcing them through a 70 μm cell strainer. Cells were washed twice in wash buffer by centrifugation (400× *g* for 5 minutes) and resuspended in culture media (RPMI 1640 + 10% FCS + 2 mM L‐Glutamine + 100 U/ml penicillin + 100 μg/ml streptomycin). Total cell number per cMLN was quantified by automatic counting on a CASY cell counter. For ex vivo stimulation cells were adjusted to 5 × 10^6^ cells/ml and seeded into 96 well plates (200 μl/well). Cells were then incubated with 50 μg/ml of TmES for 36 hours at 37°C 5% CO_2_. The culture supernatant was collected following incubation and stored at −20°C prior to assaying.

To quantify cytokine (IFNγ, IL‐5, IL‐6, IL‐9, IL‐10, IL‐13, IL‐17A, and TNFα) secretion following ex vivo stimulation a cytometric bead array (CBA) was performed. 16.6 μl of thawed culture supernatant (and standards for each cytokine) was transferred to a round bottom 96 well plate and incubated with 16.6 μl of capture beads (0.33 μl of beads per cytokine diluted in Capture Diluent) at room temperature (RT) for 5 minutes on an orbital shaker. 16.6 μl (0.33 μl of antibody per cytokine diluted in Detection Diluent) of detection antibody was added and incubated under the same conditions for a further 60 minutes. Samples were washed in 150 μl wash buffer by centrifugation at 400× *g* for 5 minutes. The supernatant was discarded and capture beads were resuspended in 70 μl of fresh wash buffer. Samples were read using a MACSQuant Analyser 10 (Miltenyi Biotech) and quantification of the data was performed using FCAP array v3.01 using settings recommended by BD Biosciences.

### 2.4 Histology and microscopy

At autopsy the caecal tip was removed and placed in 4% Neutral Buffer Formalin (NBF) overnight before transferring to 70% ethanol for storage. Fixed caecal tissue was processed into paraffin blocks, cut into 5 μm thick slices, and mounted on glass slides. To visualize goblet cells samples were dewaxed and Periodic Acid and Schiff's reagent (PAS) stained followed by counterstaining with Mayer's Haematoxylin.

For immunofluorescence imaging of pSmad2/3 dewaxed samples underwent antigen retrieval (3 minutes boiling in citrate buffer [10 mM Citric Acid, 0.05% Tween, pH 6], followed by 20 minutes cooling at RT). Samples were then washed in PBS, blocked with 5% rabbit serum (30 minutes at RT), and incubated overnight with polyclonal anti‐Smad2/3(S467) (ab53100) 1:200 at 4°C in a humidity chamber. Samples were then washed three times with PBS and incubated with AlexaFluor 594 anti‐Rabbit (1:1000) for 2 hours at RT. Samples were then washed twice with PBS, incubated for 5 minutes with DAPI (1:1000), washed twice again with PBS and once with dH_2_O before mounting in Prolong Gold Antifade Mountant.

Imaging was performed on a Nikon Eclipse Ci Upright Microscope and image analysis was undertaken in ImageJ.

### 2.5 Quantitative PCR

Caecal tissue for gene expression analysis was collected into 1 ml TRIzol™ Reagent and immediately frozen on dry ice. Samples were stored at −80°C prior to RNA extraction. RNA extraction was performed as per the TRIzol™ manufacturer's instructions. RNA concentration was calculated on a Nanodrop ND‐1000. A cDNA library was generated from 2 μg of RNA per sample using the Promega GoScript reverse transcriptase kit as per the manufacturer's instructions. The qPCR reaction was performed using the SensiFAST SYBR Hi‐ROX kit. The reaction mixture consisted of 10 μl 2x SensiFAST SYBR Hi‐ROX mix, 0.8 μl 10 μM forward primer, 0.8 μl 10 μM reverse primer, 6.4 μl RNase‐free H2O, and 2 μl cDNA. The reaction was heat cycled under the following steps: (i) 95°C 2 minutes, (ii) [95°C 5 seconds, 60°C 10 seconds, 72°C] × 40, (iii) 95°C 15 seconds (iv) 60°C 1 minute, (v) 95°C 15 seconds and (vi) 10°C hold. Gene expression level was semi‐quantified using the 2^‐δCT method with β‐actin as the house‐keeping reference gene. RNA expression is therefore given as expression level relative to β‐actin. Primer sequences are as follows: β‐actin Fwd—5′ TCT TGG GTA TGG AAT GTG GCA; β‐actin Rev 5′ ACA GCA CTG TGT TGG CAT AGA GGT; *Retnlb* Fwd—5′ GCT CTT CCC TTT CCT TCT CCA; *Retnlb* Rev—5′ AAC ACA GTG TAG GCT TCA TGC TGT.

### 2.6 Assessment of parasite length and fecundity

Individual 33‐day‐old male and female worms were isolated from mice at autopsy. To measure fecundity, individual female *Tm* worms were placed in wells in 48 well plates in 1 ml culture media (RPMI + 100 U/ml penicillin + 100 μg/ml streptomycin) and incubated at 37°C for 4 hours. At the end of incubation the total volume of culture media was collected and the number of eggs present was counted by eye under dissecting microscope. To measure size, worms were fixed in Bles medium^31^ and mounted on glass slides in PERTEX mountant. Slides were imaged using a Leica Steromicroscope at 7× magnification. Individual worm lengths were measured in ImageJ.

### 2.7 Data analysis

Statistical analysis was performed in either GraphPad prism version 9.2.0 or RStudio version 1.3.1093. Graphs were produced using the ggplot2 package in RStudio.

## 3 RESULTS

### 3.1 Co‐infection with *H. bakeri* impairs expulsion of high‐dose *T. muris* infection

**FIGURE 1:**
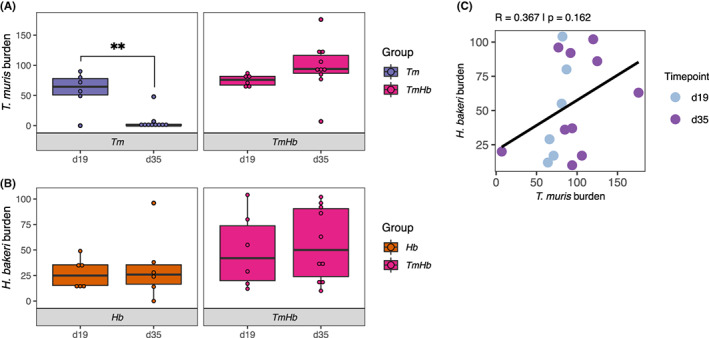
Adult worm burden during *T. muris* and *H. bakeri* co‐infection. Female C57BL/6 mice were infected with a high dose of *T. muris* (*Tm*, 200 eggs) alone or co‐infected with a high dose of *H. bakeri* (*TmHb*, 200 *Tm* eggs + 200 L3 *Hb* larvae), a high‐dose *Hb* alone group was included as a control. Parasite burden was assessed at 19 (d19) and 35 (d35) post infection for both (A) *Tm* in the caecum (*Tm* d19 *n* = 6, d35 *n* = 10; *TmHb* d19 *n* = 6, d35 *n* = 10) and (B) *Hb* in the small intestine (*Hb* d19 *n* = 6, d35 *n* = 6; *TmHb* d19 *n* = 6, d35 *n* = 10). Box plots indicate median and interquartile range, whiskers indicate 1.5× the quartile limit, points indicate individual replicates. ** Indicates adjusted *P* < .01 as calculated by Holm‐Šídák's multiple‐comparisons test. (C) Scatter plot of *Tm* burden plotted against *Hb* burden in co‐infected mice, points indicate individual replicates. Pearson's correlation coefficient (R) and *P* value was calculated using the ggcorrplot package in RStudio

To understand the impact of *Hb* co‐infection on expulsion kinetics of *Tm* we infected female C57BL/6 mice with either a high dose of *Tm* (200 embryonated eggs) alone or coinfected with a *Hb* high dose (200 L3 larvae) (*TmHb*), a high dose *Hb* alone group was included as a control. Mice were euthanized at d19 and d35 post‐infection and the *Tm* and *Hb* worm burdens in the caecum and small intestines, respectively, were assessed at autopsy. Mice that were mono‐infected with *Tm* efficiently expelled their infections by d35 with a significant drop in total worm burden between d19 and d35. Co‐infected mice by contrast were unable to expel their infection showing no reduction in *Tm* burden between the two timepoints (Figure 1A). We observed no effect of co‐infection on *Hb* burden at either timepoint relative to *Hb* mono‐infected controls (Figure 1B). Interestingly we could not detect a significant correlation between *Tm* and *Hb* worm burdens in co‐infected mice (Figure 1C) indicating that impairment of *Tm* expulsion was not dose‐dependent.

### 3.2 Co‐infection with *H. bakeri* reduces colonic MLN cellularity but does not impair parasite‐specific cytokine production

**FIGURE 2:**
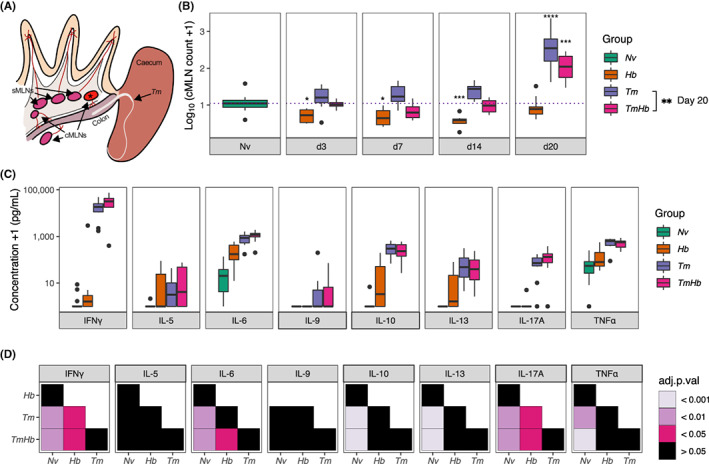
Cellularity but not cytokine responsiveness of the colonic draining lymph node proximal to the caecum is impaired during co‐infection. Female C57BL/6 mice were infected with a high dose of *T. muris* (*Tm*, 200 eggs) alone or co‐infected with *H. bakeri* (*TmHb*, 200 *Tm* eggs +200 L3 *Hb* larvae). A *H. bakeri* alone (*Hb*, 200 L3 *Hb* larvae) and uninfected (*Nv*) group were included as controls. Lymphocytes from the colonic draining mesenteric lymph node (cMLN) proximal to the caecum were isolated at d0, d3, d7, d14 and d20 post‐infection. (A) Diagrammatic representation of the anatomical position of the isolated cMLN (★) relative to the small intestine, caecum, large intestine and other MLNs. (B) Enumeration of the total number of cMLN cells transformed using a Log_10_(total cell count/1 × 10^6^) + 1) transformation. The dotted line represents the median of the *Nv* group. *Nv n* = 17, *Hb n* ≥ 4 mice/timepoint, *Tm n* ≥ 7 mice/timepoint, *TmHb n* ≥ 7 mice/timepoint. Boxes indicate the median and interquartile range, whiskers indicate 1.5× the quartile limit, dots indicate individual outlier values >1.5× the quartile limit. *s above boxes represent comparisons to the *Nv* group calculated by Dunn's multiple comparisons on untransformed data. * adjusted *P* < .05, ** adjusted *P* < .01, *** adjusted *P* < .001, **** adjusted *P* < .0001. (C) cMLN lymphocytes isolated on d20 were cultured for 36 hours in the presence of 50 μg/ml *Tm* excretory/secretory antigen and the concentration of cytokines in the supernatant was measured by cytometric bead array. *Nv n* = 13, *Hb n* = 8, *Tm n* = 10, *TmHb n* = 10. Cytokine concentration is expressed as pg/ml +1. Boxes indicate the median and interquartile range, whiskers indicate 1.5× the quartile limit, dots indicate individual outlier values >1.5× the quartile limit. For all cytokines measured, under media‐only conditions, cytokine levels were below the limit of detection for the assay. (D) Tile plot of statistical comparisons between infection groups for each cytokine. Tile colour indicates adjusted *P* value as calculated using untransformed values by pairwise Mann–Whitney tests and adjusted by Holm correction

To investigate whether there was an immune component to the loss of resistance to *Tm* infection in co‐infected mice we isolated the cMLN most proximal to the caecum (Figure 2A) from naive mice and infected groups at d3, d7, d14, and d20 post‐infection and enumerated the total number of cells present. In our experience this cMLN undergoes the most pronounced hyperplasia following *Tm* infection, suggesting it as the primary draining MLN for the caecum and/or proximal colon, this is supported by observations from other groups which demonstrate different regions of the intestines drain to specific MLNs.^30^, ^32^ As early as d3, mice mono‐infected with *Tm* displayed increased cellularity of the cMLN relative to naïve mice, with this becoming especially evident at d20 (Figure 2B). By contrast, co‐infected mice showed no‐increased cellularity of the cMLN at d3, d7, or d14, and whilst they significantly increased the total number of cMLN cells at d20, relative to uninfected controls, they had a significantly lower cell count than the *Tm* mono‐infected group. Interestingly, mice mono‐infected with *Hb* showed significantly reduced cMLN cellularity compared to uninfected controls, indicating that *Hb* infection is apparently reducing the available lymphocyte pool in the cMLN (Figure 2B).

Given the reduced cellularity in the cMLN in co‐infected mice, we next sought to identify whether co‐infection would affect the capacity of cMLN cells to produce cytokine in response to parasite antigen. In single high‐dose *Tm* infection in C57BL/6 mice, peak MLN cytokine responses occur between d19 and d21.^33^ To measure the *Tm*‐specific cytokine response, we isolated lymphocytes from the cMLN our infected groups at d20 and cultured them ex vivo in the presence of TmES (50 μg/ml). The concentration of secreted cytokines (IFNγ, IL‐5, IL‐6, IL‐9, IL‐10, IL‐13, IL‐17A, & TNFα) was measured by CBA. cMLN lymphocytes from the *Tm* and *TmHb* groups significantly increased secretion of IFNγ, IL‐6, IL‐10, IL‐13, IL‐17A, and TNFα relative to the uninfected group in response to stimulation with TmES (Figure 2C,D). As expected, *Hb* mono‐infected mice showed no difference in the production of any cytokine in response to TmES relative to naïve mice (Figure 2C,D). Interestingly we could not observe a statistical difference in the concentration of any measured cytokine between the *Tm* and *TmHb* groups (Figure 2D) indicating that co‐infection did not inhibit the cytokine responsiveness of cMLN lymphocytes following *Tm*‐specific antigen stimulation in vitro.

### 3.3 Caecal mucosal responses are maintained during co‐infection

During high‐dose *Tm* infection parasite expulsion pre‐patency is mediated by physiological changes at the caecal mucosal barrier, particularly goblet cell hyperplasia, accelerated epithelial cell turnover, and upregulation of anti‐helminth gene expression between d19 and d35 post infection.^21^, ^23^ To assess whether co‐infection was impairing these responses, caecal tissue was collected at autopsy for histology and qPCR analysis.

**FIGURE 3:**
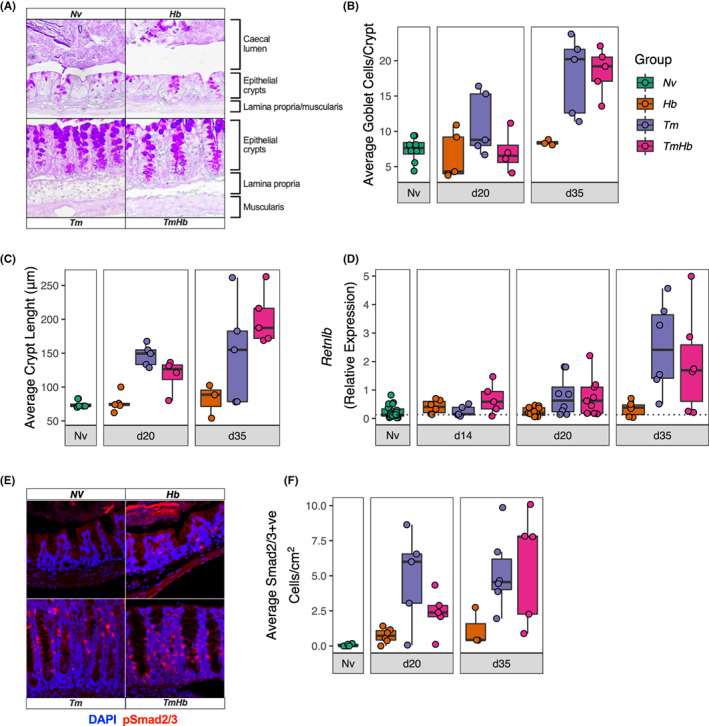
Caecal barrier responses are maintained during co‐infection despite impaired parasite expulsion. Female C57BL/6 mice were infected with a high dose of *T. muris* (*Tm*, 200 eggs) alone or co‐infected with *H. bakeri* (*TmHb*, 200 *Tm* eggs +200 L3 *Hb* larvae). A *H. bakeri* alone (*Hb*, 200 L3 *Hb* larvae) and uninfected (*Nv*) group were included as controls. Caecal tissue was collected at d14, d20 and d35 post infection for gene expression and/or histological analysis. (A) Representative images of periodic acid Schiff stained caecal epithelium from d35. Goblet cells are visible as purple circles. (B) Enumeration of goblet cell number given as the average number of goblet cells per caecal crypt (mean of 10 crypts/mouse). (C) Enumeration of the average crypt length in μm measured from the base of the crypt to the caecal lumen (mean of 10 crypts/mouse). (B and C, Nv *n* = 8; *Hb* d20 *n* = 5, d35 *n* = 3, *Tm* d20 *n* = 5, d35 *n* = 5; *TmHb* d20 *n* = 4, d35 *n* = 5) (D) Caecal expression of *Retnlb* measure by qPCR. Values were given as expression relative to β‐Actin, calculated using the 2^‐δCT method (*Nv n* = 23; *Hb* d14 *n* = 6, d20 *n* = 11, d35 *n* = 6; *Tm* d14 *n* = 5, d20 *n* = 8, d35 *n* = 6; *TmHb* d14 *n* = 5, d20 *n* = 9, d20 *n* = 6). (E) Representative immunofluorescence images of anti‐pSmad2/3 (red) staining in caecal tissue with DAPI (blue) to stain nuclei from mice at d35. (F) Enumeration of the number of pSmad2/3^+^ cells per cm^2^ of caecal tissue. Boxes represent the median and interquartile range, whiskers indicate 1.5× the quartile limit, dots indicate individual replicates (Nv *n* = 5; *Hb* d20 *n* = 6, d35 *n* = 3, *Tm* d20 *n* = 5, d35 *n* = 6; *Tm Hb* d20 *n* = 5, d35 *n* = 5)

Caecal tissue was PAS stained to visualize mucosal epithelial structure (Figure 3A). Compared to *Nv* mice (and *Hb* mice), *Tm* infected mice displayed a robust goblet cell hyperplasia (Figure 3B) and an increase in caecal crypt length (Figure 3C). We observed no statistical difference in the ability of co‐infected mice to induce these changes relative to *Tm* mono‐infected mice (Figure 3B,C). *Retnlb* (the gene responsible for resistin‐like molecule β [RELMβ] production) is a marker of type 2 polarization at mucosal barriers and RELMβ has been implicated as an anthelminthic effector molecule against *Hb*.^34^, ^35^, ^36^ We measured *Retnlb* expression in the caecum by qPCR. Both *Tm* and *TmHb* infected mice were able to upregulate *Retnlb* expression by d35 post‐infection and there was no statistical difference between these groups (Figure 3D). These data indicated that co‐infection does not prevent the development of canonical anti‐*Trichuris* effector mechanisms at the mucosal barrier.

*Hb* has been shown to secrete immunomodulatory peptides, which can dampen type 2 immune responses^37^ including a TGFβ mimic.^27^ To determine whether *Hb* infection could induce TGFβ signalling in the caecal mucosa we performed immunofluorescence staining against pSmad2/3 (Figure 3E). Smad2/3 becomes phosphorylated during canonical TGFβ signalling^38^ and, therefore, pSmad2/3 can be used as a proxy‐measurement for activation of this pathway. *Hb* mono‐infected mice showed modest increases in the number of pSmad2/3^+^ cells in the caecum relative to uninfected mice at both d20 and d35 post‐infection (Figure 3F). However, *Tm* mono‐infected mice showed greater numbers of pSmad2/3^+^ cells than uninfected and *Hb* mice, and co‐infection did not alter this (Figure 3F).

### 3.4 Co‐infection does not impair immunization‐induced anti‐*Trichuris muris* immunity

**FIGURE 4:**
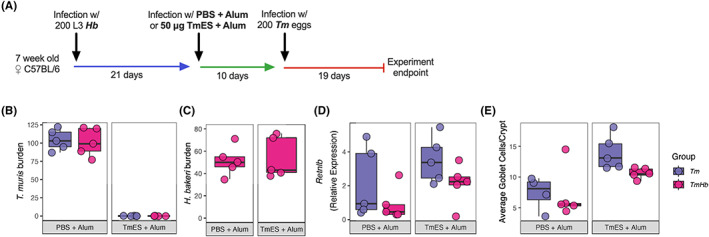
Infection with *H. bakeri* does not impair immunization‐induced anti‐*T. muris* immunity. (A) Schematic representation of experimental design. Five Female C57BL/6 mice per group were infected with 200 L3 *H. bakeri* (*Hb*) larvae and allowed 3 weeks to establish a chronic infection. Mice were then immunized with 50 μg of *T. muris* (*Tm*) excretory/secretory antigen (TmES) in Alum or given a PBS + Alum control dose. 10 days later mice were orally gavaged with 200 *Tm* eggs. Mice were culled at d19 post *Tm* infection. Total (B) *Tm* and (C) *Hb* total worm burdens were assessed at autopsy. (D) Relative expression of *Retnlb* in the caecum was measured by qPCR. *Retnlb* level is given relative to β‐actin as calculated by 2^‐δCT. (E) Enumeration of goblet cell number given as the average number of goblet cells per caecal crypt (mean of 10 crypts/mouse). Boxes represent the median and interquartile range, whiskers indicate 1.5× the quartile limit, dots indicate individual replicates

We next sought to determine whether infection with *Hb* would impair the efficacy of immunization against *Tm*. To do this we used a previously published immunization model^39^ in which parenteral injection of TmES is sufficient to induce near‐sterile immunity to a high‐dose *Tm* infection given 10 days later with almost complete expulsion of parasites by d21 post infection. Here, we first infected mice with a high dose of *Hb* (200 L3 larvae) and allowed 21 days for a chronic infection to establish. Mice were then immunized with a single dose of 50 μg of TmES in Alum (or given a PBS + Alum control injection). 10 days later mice were given a high dose of (200) *Tm* eggs. At d19 post *Tm* infection mice were euthanized and parasite burden was assessed (Figure 4A). As expected, immunized mice which were not previously infected with *Hb* efficiently expelled *Tm* by d19 compared to mice which had received a sham immunization. Interestingly, mice previously infected with *Hb* showed no defect in immunization‐induced protective immunity (Figure 4B). Immunization against *Tm* had no effect on *Hb* worm burden (Figure 4C). When we measured markers of type 2 immunity in the caecum enhanced in immunized mice, *Retnlb* expression (Figure 4D) and goblet cell hyperplasia (Figure 4E), we found that concurrent infection with *Hb* did not limit the induction of these responses.

### 3.5 Female *Trichuris muris* growth is enhanced during co‐infection in immune‐deficient mice

**FIGURE 5:**
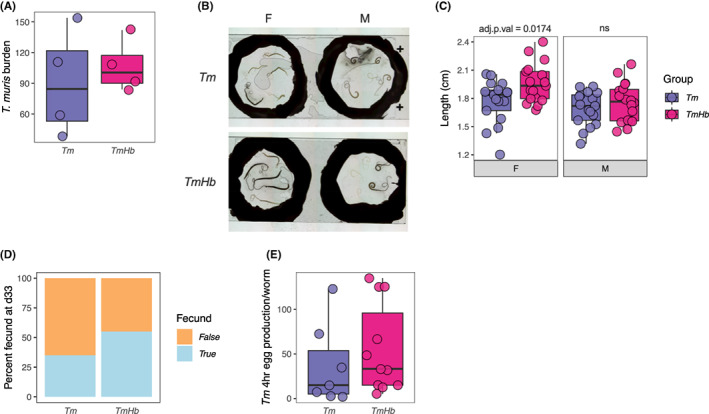
Co‐infection with *H. bakeri* increases female *Tm* length in immunodeficient mice. Female severe combined immunodeficient (SCID) mice were infected with a high dose (200) of *T. muris* eggs either alone (*Tm*) or combined with a high dose of *H. bakeri* (200 L3 larvae) (*TmHb*). Mice were euthanized at d33 post‐infection and *Tm* worms were isolated from the caecum at autopsy. (A) Total *Tm* worm burden. (B) Representative images of female [F] and male [M] worms isolated from *Tm* and *TmHb* infected mice. (C) Quantification of total worm length in cm for female and male worms. 4–5 worms of each sex were collected from each mouse and measured in ImageJ. (D) Proportion of female worms producing eggs (fecund) in 4 hours ex vivo culture at d33 in *Tm* and *TmHb* groups, *n* = 20 worms/group. (E) Quantification of the total number of eggs produced in 4 hours ex vivo culture for fecund worms. For box plots, boxes represent the median and interquartile range, whiskers indicate 1.5× the quartile limit, dots indicate individual replicates. Adjusted *P* value (adj.*P*.val) was calculated by Holm‐Šídák's multiple‐comparisons test

Given that *Hb* co‐infection dramatically impaired expulsion of *Tm* but did not significantly affect canonical drivers (IL‐13) or effectors (goblet cell hyperplasia, increased crypt length, type 2 gene expression) of anti‐*Tm* immunity, we aimed to identify if *Hb* was influencing *Tm*′s ability to establish an infection independent of any effect *Hb* may exert on adaptive immunity. To do this we infected female SCID mice, which lack mature T and B cells and are highly susceptible to *Tm* infection, with a high dose of *Tm* alone or co‐infected them with *Hb*. Total *Tm* burden was assessed at autopsy. We observed no statistical difference in the total number of *Tm* present between mono‐infected and co‐infected mice (Figure 5A). 4–5 worms of each sex per mouse were fixed on glass slides and imaged by light microscopy. Female *Tm* worms isolated from co‐infected were visibly larger than those from mono‐infected mice (Figure 5B), and when we quantified their lengths we found them to be significantly longer (Figure 5C). Additionally, a greater proportion of the female worms isolated from co‐infected mice were fecund (Figure 5D) and, in a 4 hours period in ex vivo culture, there was a trend indicating female worms form co‐infected mice shed on average a greater number eggs (Figure 5D) than worms isolated from mono‐infected mice, although this did not reach statistical significance. We did not observe a difference in the length of male worms collected from these mice.

## 4 DISCUSSION

Helminth‐helminth co‐infection in humans is associated with increased parasite burden for both infecting species and poorer health outcomes, however, it is not understood whether this is correlative (i.e., individuals susceptible to one species are inherently susceptible to other species) or if there is a causative relationship where one GI helminth renders individuals more susceptible to other infections. Here, we used a model of *Hb* and *Tm* co‐infection in C57BL/6 mice to investigate the consequences of co‐infection on immune responses to *Tm*. We found that co‐infection rendered mice that would otherwise be resistant to high dose *Tm* infection highly susceptible. This was associated with reduced cellularity in the cMLN draining the site of infection, however, cytokine responsiveness in these cells, and physiological intestinal barrier responses associated with *Tm* expulsion was not significantly impaired. Also we show that this co‐infection induced susceptibility does not impair parenteral immunization‐induced immunity to *Tm* infection. Finally, we provide evidence that co‐infection can enhance *Tm* growth and fecundity independent of *Hb* effects on adaptive immunity.

The complete loss of resistance to high‐dose *Tm* infection in C57BL/6 mice as a result of co‐infection is a striking phenotype, here made even more striking by the lack of significant reductions in the major canonical mechanisms associated with expulsion of worms in this model both in the intestinal tissue and draining lymph node. Th2‐derived IL‐13 is the cornerstone of resistance to *Tm*,^15^, ^22^ it drives the primary effector mechanisms through which *Tm* worms are physically expelled into the lumen, these include goblet cell hyperplasia, accelerated epithelial cell turnover, and re‐composition of the mucin barrier.^21^, ^23^ Here, however, we observe comparable IL‐13 secretion from cMLN lymphocytes in response to parasite antigen in vitro between mono‐infected and co‐infected mice. Consistent with this, we could not detect statistically significant differences in the caecal mucosal response between *Tm* and *TmHb* infected mice. This indicated that *Tm* was persisting in co‐infected mice despite apparently functioning anti‐*Tm* mechanisms. How this is mediated remains unclear. Given that we only examined specific timepoints (those previously associated with peak cytokine and barrier response) it may be that defects in key immune responses are occurring earlier in infection than expected, but that their effects are potentiated later in infection. Additionally, increased replicates may have revealed subtle differences in the caecal response between groups at the timepoints measured not detected in this study. It may be that, rather than inducing a single failure point in the caecal response, that infection with *Hb* causes subtle reductions in a range of anti‐*Tm* effector mechanisms, the cumulative weight of which results in a failure to expel the parasite. Furthermore, deeper immune phenotyping ‐ for example flow cytometric analysis of T cell subsets, measurement of the expression of a broader range of Th2‐associated genes, or cytokine measurement in the caecum ‐ may reveal defects in aspects of the Th2 immune response not assessed here.

Previous work has shown that infection with *Hb* alone has a significant effect on the cellularity of peripheral lymph nodes including inguinal, axillary and brachial lymph nodes by forcing a redistribution of the limited lymphocyte pool from these lymph nodes to the small intestinal MLN (sMLN).^40^ Consistent with this, we observed atrophy of the cMLN in mice mono‐infected with *Hb* and a reduced capacity to expand the cMLN lymphocyte pool in co‐infected mice relative to mice infected with *Tm* alone. *Hb*‐triggered reduction of peripheral draining LN cellularity has previously been associated with impaired immune responses to *Bacille Calmette*‐*Guerin* (BCG) and influenza A virus, and in the case of BCG reversal of draining LN atrophy was sufficient to restore the BCG‐induced immune response.^40^, ^41^ This supports a hypothesis through which, rather than directly suppressing immune responses to *Tm*, *Hb* outcompetes *Tm* in attracting a limited pool of circulating lymphocytes to the sMLN thus delaying the expansion of *Tm*‐specific Th cells. As *Tm* is a large multi‐cellular parasite immune responses that target it, including dramatic physiological barrier changes, are most effective against early larval stages. Even small delays in the activation of anti‐*Tm* effector mechanisms may be sufficient to allow *Tm* to mature to a size at which these mechanisms become ineffective at manual expulsion of the worms. This would explain the presence of adult parasites at d35 despite a seemingly intact barrier immune response.

The gold standard for long‐term protection against pathogens is vaccination. However, there is concern that in areas endemic with multiple‐parasite species, particularly helminths with the capacity to immunosuppress,^37^ that vaccination efforts against one species may be compromised by infection with another. Indeed, there is evidence that infection with helminths can limit efficacy of vaccination against infections.^16^, ^17^ However, as there is no existing vaccine that targets human helminths it is unclear whether this will be a significant problem for future anti‐helminth vaccination efforts. Encouragingly, in our immunization model, we did not observe a defect in immunization‐induced immunity to *Tm* as a consequence of concurrent infection with *Hb*. This might indicate that *Hb* promotes *Tm* survival though mechanisms not linked with those associated with immunization‐induced immunity, which are thought to target very early larval stages (between d0‐d14),^39^ whereas effector mechanisms responsible for *Tm* expulsion that develop during infection act on more mature stages of the parasite (between d19 and d35).^42^ However, it is unclear whether there exist distinct mechanisms that target early vs late larval stages. It is perhaps more likely that early larval stages are simply more susceptible to canonical anti‐*Tm* effector mechanisms and that subtle reductions/delays caused by *Hb* are insufficient to overcome this susceptibility. This supports the idea that mechanisms of *Tm* expulsion are dependent on the rapidity with which they can be induced.

In our initial experiments, we presupposed that that *Hb*'s capacity to impair resistance to *Tm* infection would operate through immunosuppressive mechanisms, this hypothesis was driven by an abundance of data demonstrating that *Hb*‐derived peptides can directly suppress type 2 immune responses.^28^, ^37^ However, when we either mono‐infected or co‐infected SCID mice, we observed that female *Tm* were physically larger in the co‐infected mice and were on average more fecund. Interestingly, we found similar total numbers of parasites between infection groups indicating that *Hb* infection did not enhance the establishment of L1 *Tm* larvae in the epithelium, but could promote parasite growth and fecundity independent of its effects on manipulating the adaptive immune response. How this occurs is unclear, but a potential explanation would be through *Hb*'s known effects on the composition of the intestinal bacterial microbiota.^43^, ^44^ A *Hb* recomposed microbiota may favour *Tm* maturation through increased abundance of specific metabolites. Indeed, metabolites produced by the microbiota during *Hb* infection are known to act directly on their host^43^ and it is conceivable that these effects may have bystander consequences for *Tm*. Alternatively, *Tm* may sense factors secreted by *Hb* and respond by increasing the rate at which they reach maturity and complete their lifecycle and guard against potential loss of host fitness indicated by polyparasitism. However, SCID mice retain a functional innate immune system, and whilst adaptive‐driven mechanisms are thought to be principally responsible for *Tm* expulsion, we cannot rule out *Hb* co‐infection effects of female *Tm* being mediated via innate immunity. It will be interesting in future studies to interrogate whether *Tm* growth and fecundity is altered by co‐infection in immune‐competent mice, experimentally this could be achieved by using a low‐dose *Tm* infection model in which *Tm* induces an ineffective Th1 response allowing the infection to reach chronicity.

In summary, our data provide a characterization of the immune phenotype during a model helminth‐helminth co‐infection. We propose that there is a mechanistic basis to the higher parasite burdens observed in co‐infected individuals and that this can be independent of the adaptive immune response. Finally, our data present the exciting possibility of helminth‐helminth communication in the GI system with consequences for parasite survival and growth.

## AUTHOR CONTRIBUTIONS

Stefano A. P. Colombo designed and conducted the work presented here, performed the data analysis, and drafted this manuscript. Seona Thompson contributed significantly to the experimental work and provided feedback on this manuscript. Allison J. Bancroft contributed to the experimental work and preparation of this manuscript. Richard K. Grencis contributed to the design and direction of the project and contributed to the preparation of this manuscript.

## FUNDING INFORMATION

Supported by the Wellcome Trust, United Kingdom 103132/Z/13/Z/WT, Z10661/Z/18/Z/WT, 203128/Z/16/Z/WT

## CONFLICT OF INTEREST

The authors declare no conflicts of interest.

## ANIMAL STUDY APPROVAL STATEMENT

All animal work was conducted under HO licence number P043A3082 following authorization by the local AWERB committee. The chairman of the AWERB committee is Professor John McLaughlin. Contact details are: phone: +441612064363. Email: john.mclaughlin@manchester.ac.uk

### PEER REVIEW

The peer review history for this article is available at https://publons.com/publon/10.1111/pim.12936.

## Supporting information


**Table S1** Worm burden raw data for Figure 1A–C.
**Table S2**. Raw data for Figure 2B. cMLN counts N^o^ cells/cMLN (×10^6^)
**Table S3**. Raw data for Figure 2B. Cytokine concentration (pg/ml)
**Table S4**. Raw data for Figure 3B,C. Quantification of caecal histology
**Table S5**. Raw data for Figure 3D. Relative expression of *Retnlb* normalized to β‐actin expression
**Table S6**. Raw data for Figure 3F. Count of Smad2/3+ve cells/cm^2^
**Table S7**. Raw data for Figure 4
**Table S8**. Raw data for Figure 5A. Worm burdens (N^o^ Tm worms/mouse)
**Table S9**. Raw data for Figure 5C. Worm lengths in cm
**Table S10**. Raw data for Figure 5D. Percent of fecund female worms/mouse
**Table S11**. Raw data for Figure 5E.

## Data Availability

The data that supports the findings of this study are available in the supplementary material of this article.
