# Supplementary material for: Anti‐Trichuris mucosal responses are maintained during H. bakeri co‐infection despite impaired parasite expulsion
Source: Parasite Immunol. 2022 May 31;44(8):e12936. doi: 10.1111/pim.12936 (PMC9542692; doi:10.1111/pim.12936)
Supplement: Supplementary file 1 — Table S1 Worm burden raw data for Figure 1A–C. Table S2. Raw data for Figure 2B. cMLN counts No cells/cMLN (×106) Table S3. Raw data for Figure 2B. Cytokine concentration (pg/ml) Table S4. Raw data for Figure 3B,C. Quantification of caecal histology Table S5. Raw data for Figure 3D. Relative expression of Retnlb normalized to β‐actin expression Table S6. Raw data for Figure 3F. Count of Smad2/3+ve cells/cm2 Table S7. Raw data for Figure 4 Table S8. Raw data for Figure 5A. Worm burdens (No Tm worms/mouse) Table S9. Raw data for Figure 5C. Worm lengths in cm Table S10. Raw data for Figure 5D. Percent of fecund female worms/mouse Table S11. Raw data for Figure 5E. [file PIM-44-e12936-s001.docx]

**Supplementary Table 1.**

| **Timepoint** | **Group** | **TmBurden** | **HbBurden** |
| --- | --- | --- | --- |
| d19 | Tm | 80 | 0 |
| d19 | Tm | 57 | 0 |
| d19 | Tm | 49 | 0 |
| d19 | Tm | 90 | 0 |
| d19 | Tm | 72 | 0 |
| d19 | Tm | 0 | 0 |
| d35 | Tm | 0 | 0 |
| d35 | Tm | 7 | 0 |
| d35 | Tm | 0 | 0 |
| d35 | Tm | 3 | 0 |
| d35 | Tm | 48 | 0 |
| d35 | Tm | 0 | 0 |
| d35 | Tm | 0 | 0 |
| d35 | Tm | 0 | 0 |
| d35 | Tm | 0 | 0 |
| d35 | Tm | 0 | 0 |
| d19 | TmHb | 66 | 29 |
| d19 | TmHb | 64 | 12 |
| d19 | TmHb | 71 | 17 |
| d19 | TmHb | 87 | 80 |
| d19 | TmHb | 81 | 55 |
| d19 | TmHb | 82 | 104 |
| d35 | TmHb | 106 | 17 |
| d35 | TmHb | 94 | 10 |
| d35 | TmHb | 7 | 20 |
| d35 | TmHb | 85 | 36 |
| d35 | TmHb | 125 | 86 |
| d35 | TmHb | 77 | 96 |
| d35 | TmHb | 92 | 92 |
| d35 | TmHb | 176 | 63 |
| d35 | TmHb | 94 | 37 |
| d35 | TmHb | 120 | 102 |
| d19 | Hb | 0 | 15 |
| d19 | Hb | 0 | 16 |
| d19 | Hb | 0 | 13 |
| d19 | Hb | 0 | 49 |
| d19 | Hb | 0 | 34 |
| d19 | Hb | 0 | 36 |
| d35 | Hb | 0 | 14 |
| d35 | Hb | 0 | 38 |
| d35 | Hb | 0 | 0 |
| d35 | Hb | 0 | 28 |
| d35 | Hb | 0 | 24 |
| d35 | Hb | 0 | 96 |

**Worm burden raw data for figure 1A-C.**

**Supplementary Table 2.**

**Raw data for Figure 2B. cMLN counts N^o^ cells/cMLN (x10^6^)**

| **Group** | **Timepoint** | **cMLN cells** |
| --- | --- | --- |
| Nv | Nv | 2.07 |
| Nv | Nv | 3.87 |
| Nv | Nv | 1.55 |
| Nv | Nv | 2.1 |
| Nv | Nv | 1.74 |
| Nv | Nv | 1.56 |
| Nv | Nv | 1.35 |
| Nv | Nv | 1.84 |
| Nv | Nv | 0.82 |
| Nv | Nv | 1.43 |
| Nv | Nv | 1.61 |
| Nv | Nv | 2.36 |
| Nv | Nv | 2.14 |
| Nv | Nv | 1.804 |
| Nv | Nv | 1.92 |
| Nv | Nv | 2.012 |
| Nv | Nv | 2.292 |
| Tm | d3 | 2.24 |
| Tm | d3 | 3.14 |
| Tm | d3 | 3.21 |
| Tm | d3 | 1.61 |
| Tm | d3 | 3.75 |
| Tm | d3 | 2.33 |
| Tm | d3 | 0.69 |
| Tm | d7 | 3.85 |
| Tm | d7 | 4.26 |
| Tm | d7 | 1.9 |
| Tm | d7 | 1.34 |
| Tm | d7 | 2.43 |
| Tm | d7 | 2.1 |
| Tm | d7 | 3.15 |
| Tm | d14 | 4.33 |
| Tm | d14 | 3.2 |
| Tm | d14 | 2.03 |
| Tm | d14 | 2.3 |
| Tm | d14 | 1.97 |
| Tm | d14 | 3.24 |
| Tm | d14 | 3.46 |
| Tm | d14 | 3.62 |
| Tm | d20 | 4.08 |
| Tm | d20 | 5.03 |
| Tm | d20 | 11.24 |
| Tm | d20 | 6.19 |
| Tm | d20 | 14.41 |
| Tm | d20 | 17.87 |
| Tm | d20 | 11.74 |
| Tm | d20 | 7.97 |
| Tm | d20 | 27.55 |
| Tm | d20 | 13.432 |
| Tm | d20 | 15.352 |
| Tm | d20 | 17.992 |
| Tm | d20 | 9.5 |
| Hb | d3 | 1.06 |
| Hb | d3 | 1.39 |
| Hb | d3 | 0.69 |
| Hb | d3 | 1.48 |
| Hb | d3 | 0.65 |
| Hb | d7 | 1.23 |
| Hb | d7 | 0.51 |
| Hb | d7 | 0.62 |
| Hb | d7 | 1.69 |
| Hb | d14 | 1.3 |
| Hb | d14 | 0.9 |
| Hb | d14 | 0.66 |
| Hb | d14 | 0.3 |
| Hb | d14 | 0.82 |
| Hb | d14 | 0.64 |
| Hb | d14 | 0.8 |
| Hb | d20 | 3.54 |
| Hb | d20 | 2.46 |
| Hb | d20 | 0.85 |
| Hb | d20 | 1.3 |
| Hb | d20 | 0.98 |
| Hb | d20 | 1.6 |
| Hb | d20 | 1.532 |
| Hb | d20 | 1.068 |
| Hb | d20 | 1.352 |
| Hb | d20 | 1.52 |
| TmHb | d3 | 2.25 |
| TmHb | d3 | 1.65 |
| TmHb | d3 | 1.33 |
| TmHb | d3 | 1.9 |
| TmHb | d3 | 1.94 |
| TmHb | d3 | 1.67 |
| TmHb | d3 | 1.59 |
| TmHb | d3 | 1.85 |
| TmHb | d7 | 1.51 |
| TmHb | d7 | 2.24 |
| TmHb | d7 | 1.86 |
| TmHb | d7 | 0.94 |
| TmHb | d7 | 0.9 |
| TmHb | d7 | 1.43 |
| TmHb | d7 | 1.03 |
| TmHb | d7 | 0.79 |
| TmHb | d14 | 1.86 |
| TmHb | d14 | 1.18 |
| TmHb | d14 | 1.34 |
| TmHb | d14 | 1.68 |
| TmHb | d14 | 2.35 |
| TmHb | d14 | 1.08 |
| TmHb | d14 | 2.29 |
| TmHb | d20 | 3.59 |
| TmHb | d20 | 5.89 |
| TmHb | d20 | 5.51 |
| TmHb | d20 | 7.55 |
| TmHb | d20 | 3.36 |
| TmHb | d20 | 8.84 |
| TmHb | d20 | 6.71 |
| TmHb | d20 | 10.04 |
| TmHb | d20 | 10.44 |
| TmHb | d20 | 4.74 |
| TmHb | d20 | 10.712 |
| TmHb | d20 | 9.224 |
| TmHb | d20 | 4.508 |

**Supplementary Table 3.**

**Raw data for Figure 2B. Cytokine concentration (pg/mL)**

| Group | IFN | IL5 | IL6 | IL9 | IL10 | IL13 | IL17A | TNF |
| --- | --- | --- | --- | --- | --- | --- | --- | --- |
| Nv | 4.30638781 | 0 | 15.033026 | 0 | 0 | 0 | 0 | 71.4465485 |
| Nv | 7.731179 | 0 | 17.9458794 | 0 | 0 | 0 | 0 | 124.304844 |
| Nv | 0 | 0 | 0 | 0 | 0 | 0 | 0 | 0 |
| Nv | 0 | 0 | 21.8868913 | 0 | 0 | 0 | 0 | 250.763352 |
| Nv | 0 | 0 | 33.2728246 | 0 | 0 | 0 | 0 | 121.621943 |
| Nv | 0 | 0 | 121.242093 | 0 | 0 | 0 | 0 | 82.1858381 |
| Nv | 0 | 0 | 0 | 0 | 0 | 0 | 0 | 53.3454787 |
| Nv | 0 | 0 | 135.295692 | 0 | 0 | 0 | 0 | 77.5604641 |
| Nv | 0 | 0 | 3.23696449 | 0 | 0 | 0 | 0 | 25.6065976 |
| Nv | 0 | 0 | 0 | 0 | 0 | 0 | 0 | 7.2368531 |
| Nv | 0.67592351 | 1.14494953 | 36.8512103 | 0 | 5.87493822 | 0 | 0 | 41.5099202 |
| Nv | 0.03811686 | 0 | 47.9620842 | 0 | 0 | 0 | 0 | 26.660239 |
| Nv | 0 | 0 | 19.4562127 | 0 | 0 | 0 | 0 | 20.09392 |
| Hb |  | 0 | 379.390209 | 0 | 149.238215 | 1.63151039 | 4.03849821 | 499.934233 |
| Hb | 2933.71231 | 0 | 568.716581 | 0 | 196.564806 | 0 | 0 | 557.525503 |
| Hb | 0 | 0 | 11.4670264 | 0 | 0 | 0 | 0 | 70.6061573 |
| Hb | 0 | 0 | 68.4369814 | 0 | 0 | 0 | 0 | 150.970083 |
| Hb | 0 | 0 | 593.784304 | 0 | 34.6606802 | 0 | 0 | 88.0374602 |
| Hb | 0.61047332 | 23.6844615 | 157.495504 | 0 | 0 | 25.8953468 | 0 | 53.4357016 |
| Hb | 0.67592351 | 87.2816103 | 191.932228 | 0 | 10.0654505 | 78.8703743 | 0 | 33.6071904 |
| Hb | 4.0583409 | 22.9501378 | 111.755992 | 0 | 0 | 18.8425629 | 0 | 49.7577607 |
| Tm | 2245.96322 | 5.13494191 | 435.390378 | 0 | 67.5716971 | 62.7679371 | 0 | 398.952011 |
| Tm | 23891.8313 | 21.6298024 | 474.057428 | 11.2064072 | 499.560672 | 308.696525 | 129.106516 | 622.547264 |
| Tm | 48326.9774 | 0 | 754.693875 | 0 | 341.944316 | 35.2288022 | 170.310319 | 788.826021 |
| Tm | 39332.9432 | 9.17623924 | 964.391567 | 7.61325981 | 649.581921 | 137.173821 | 154.793546 | 762.233088 |
| Tm | 22484.6717 | 0 | 1480.93469 | 0 | 563.713023 | 8.52949433 | 73.6340663 | 728.355392 |
| Tm | 25907.7798 | 0 | 921.911311 | 0 | 330.543556 | 16.7279335 | 67.9523494 | 728.355392 |
| Tm | 10613.9339 | 0 | 824.492441 | 0 | 272.317118 | 3.57416547 | 49.9033547 | 458.538686 |
| Tm | 13651.928 | 0.65688455 | 1628.26418 | 0 | 247.444259 | 17.918175 | 67.2063963 | 642.390561 |
| Tm | 15204.1506 | 8.94055515 | 1158.34712 | 0 | 155.811466 | 76.3684574 | 92.7332686 | 384.730735 |
| Tm | 1721.17274 | 42.2956985 | 172.798069 | 198.69856 | 137.695453 | 218.180764 | 9.22379036 | 87.6656351 |
| TmHb | 44495.663 | 0 | 1240.95586 | 0 | 595.380508 | 23.2680199 | 379.404723 | 865.120028 |
| TmHb | 74236.2231 | 0.81265963 | 600.234059 | 0 | 494.083719 | 39.0253064 | 166.7365 | 557.525503 |
| TmHb | 400.008738 | 45.4154544 | 201.372089 | 0 | 26.2344643 | 37.5687644 | 0 | 218.743717 |
| TmHb | 26438.2197 | 0 | 911.618532 | 0 | 194.255122 | 10.3501506 | 183.420764 | 576.202053 |
| TmHb | 73452.9546 | 8.48161899 | 1395.50499 | 0 | 569.916044 | 89.9313441 | 299.042043 | 712.062196 |
| TmHb | 38535.0356 | 0 | 1240.95586 | 0 | 319.496534 | 7.96310152 | 166.7365 | 643.673823 |
| TmHb | 17462.7619 | 0 | 1118.27324 | 0 | 278.637991 | 1.63151039 | 89.1452612 | 589.038588 |
| TmHb | 17625.0059 | 66.3781801 | 1514.99252 | 71.0362303 | 160.69113 | 236.993836 | 68.8138969 | 298.172274 |
| TmHb | 18302.8339 | 47.881699 | 1095.87451 | 11.4913728 | 105.082259 | 99.716627 | 44.6396535 | 317.788787 |
| TmHb | 39853.9138 | 74.100455 | 1912.56471 | 39.5529373 | 136.281209 | 129.741837 | 102.147564 | 432.467696 |

**Supplementary Table 4.**

**Raw data for Figure 3B&C. Quantification of caecal histology**

| **Group** | **Timepoint** | **Goblet (average/crypt)** | **Length (μm)** |
| --- | --- | --- | --- |
| Hb | d20 | 9.2 | 76 |
| Hb | d20 | 10.9 | 100.1 |
| Hb | d20 | 4.3 | 62.2 |
| Hb | d20 | 4 | 72.9 |
| Hb | d20 | 3.8 | 74.1 |
| Hb | d35 | 8.8 | 88.7 |
| Hb | d35 | 8.3 | 102.5 |
| Hb | d35 | 8.1 | 54.1 |
| Tm | d20 | 8.8 | 154.8 |
| Tm | d20 | 15.3 | 149.5 |
| Tm | d20 | 6.7 | 133.3 |
| Tm | d20 | 8 | 167.6 |
| Tm | d20 | 16.4 | 128.7 |
| Tm | d35 | 12.6 | 78.1 |
| Tm | d35 | 23.8 | 261.6 |
| Tm | d35 | 11.4 | 78.4 |
| Tm | d35 | 21.6 | 154.9 |
| Tm | d35 | 20.2 | 182.8 |
| TmHb | d20 | 6.1 | 136.4 |
| TmHb | d20 | 11.2 | 131.4 |
| TmHb | d20 | 7 | 121.5 |
| TmHb | d20 | 4.1 | 80.2 |
| TmHb | d35 | 17.1 | 168.9 |
| TmHb | d35 | 20.5 | 171.9 |
| TmHb | d35 | 19.2 | 263 |
| TmHb | d35 | 13.6 | 187.4 |
| TmHb | d35 | 22.1 | 216.3 |
| Nv | Nv | 9.4 | 72 |
| Nv | Nv | 8.1 | 72.2 |
| Nv | Nv | 7.2 | 82.9 |
| Nv | Nv | 5.6 | 70.3 |
| Nv | Nv | 4.4 | 70.4 |
| Nv | Nv | 9.4 | 72 |
| Nv | Nv | 8.1 | 72.2 |
| Nv | Nv | 7.2 | 82.9 |

**Supplementary Table 5**

**Raw data for Figure 3D. Relative expression of *Retnlb* normalised to β-actin expression**

| **Group** | **Relative Expression** | **Timepoint** |
| --- | --- | --- |
| Hb | 0.491184284 | d14 |
| Hb | 0.64223576 | d14 |
| Hb | 0.330551605 | d14 |
| Hb | 0.703634742 | d14 |
| Hb | 0.159341396 | d14 |
| Hb | 0.140882984 | d14 |
| Hb | 0.160768411 | d20 |
| Hb | 0.463283627 | d20 |
| Hb | 0.192252555 | d20 |
| Hb | 0.163592968 | d20 |
| Hb | 0.445230826 | d20 |
| Hb | 0.367956958 | d20 |
| Hb | 0.247657751 | d20 |
| Hb | 0.388499696 | d20 |
| Hb | 0.066572031 | d20 |
| Hb | 0.061106643 | d20 |
| Hb | 0.064746781 | d20 |
| Hb | 0.038903691 | d35 |
| Hb | 0.706594203 | d35 |
| Hb | 0.412282588 | d35 |
| Hb | 0.353978612 | d35 |
| Hb | 0.061200037 | d35 |
| Hb | 0.499512173 | d35 |
| Tm | 0.515988605 | d14 |
| Tm | 0.39024634 | d14 |
| Tm | 0.129134768 | d14 |
| Tm | 0.081325432 | d14 |
| Tm | 0.153951068 | d14 |
| Tm | 0.872134841 | d20 |
| Tm | 0.410479732 | d20 |
| Tm | 0.271214823 | d20 |
| Tm | 0.147035497 | d20 |
| Tm | 1.814923101 | d20 |
| Tm | 0.142660226 | d20 |
| Tm | 0.85207886 | d20 |
| Tm | 1.809510006 | d20 |
| Tm | 0.509195933 | d35 |
| Tm | 3.765574763 | d35 |
| Tm | 3.277122682 | d35 |
| Tm | 1.372233924 | d35 |
| Tm | 4.566659493 | d35 |
| Tm | 1.544298047 | d35 |
| TmHb | 1.469271568 | d14 |
| TmHb | 0.588124106 | d14 |
| TmHb | 0.947438706 | d14 |
| TmHb | 0.086062134 | d14 |
| TmHb | 0.337477726 | d14 |
| TmHb | 0.452198114 | d20 |
| TmHb | 1.100701627 | d20 |
| TmHb | 0.73971538 | d20 |
| TmHb | 0.619127839 | d20 |
| TmHb | 2.207666389 | d20 |
| TmHb | 1.18750719 | d20 |
| TmHb | 0.164098224 | d20 |
| TmHb | 0.180774942 | d20 |
| TmHb | 0.177919955 | d20 |
| TmHb | 2.866633293 | d35 |
| TmHb | 1.641857787 | d35 |
| TmHb | 4.993583007 | d35 |
| TmHb | 0.214689708 | d35 |
| TmHb | 0.253642537 | d35 |
| TmHb | 1.742126371 | d35 |
| Nv | 0.383349182 | Nv |
| Nv | 0.824332827 | Nv |
| Nv | 0.190249512 | Nv |
| Nv | 0.070239661 | Nv |
| Nv | 0.293689457 | Nv |
| Nv | 0.226583326 | Nv |
| Nv | 0.03014805 | Nv |
| Nv | 0.068145381 | Nv |
| Nv | 0.109382183 | Nv |
| Nv | 0.130949063 | Nv |
| Nv | 0.06203655 | Nv |
| Nv | 0.102348137 | Nv |
| Nv | 0.02445194 | Nv |
| Nv | 0.55606572 | Nv |
| Nv | 0.515748421 | Nv |
| Nv | 0.111521191 | Nv |
| Nv | 0.140072928 | Nv |
| Nv | 0.102348137 | Nv |
| Nv | 0.02445194 | Nv |
| Nv | 0.55606572 | Nv |
| Nv | 0.515748421 | Nv |
| Nv | 0.111521191 | Nv |
| Nv | 0.140072928 | Nv |

**Supplementary Table 6**

**Raw data for Figure 3F. Count of Smad2/3+ve cells/cm^2^**

| **Group** | **Timepoint** | **Smad2/3+ve cells** |
| --- | --- | --- |
| Hb | d20 | 1.41943298 |
| Hb | d20 | 0 |
| Hb | d20 | 0.53381733 |
| Hb | d20 | 1.1509301 |
| Hb | d20 | 0.37372699 |
| Hb | d20 | 0.92849553 |
| Hb | d35 | 2.74827342 |
| Hb | d35 | 0.408292 |
| Hb | d35 | 0.42413138 |
| Tm | d20 | 0.06206477 |
| Tm | d20 | 6.00209821 |
| Tm | d20 | 3.04887833 |
| Tm | d20 | 6.55686591 |
| Tm | d20 | 8.63772437 |
| Tm | d35 | 1.95196697 |
| Tm | d35 | 6.70189688 |
| Tm | d35 | 4.65231808 |
| Tm | d35 | 4.44733269 |
| Tm | d35 | 9.86406656 |
| Tm | d35 | 3.87767173 |
| TmHb | d20 | 0.1177163 |
| TmHb | d20 | 2.08739669 |
| TmHb | d20 | 2.38477036 |
| TmHb | d20 | 2.88906009 |
| TmHb | d20 | 4.33570765 |
| TmHb | d35 | 7.82465477 |
| TmHb | d35 | 10.0848811 |
| TmHb | d35 | 0.88543216 |
| TmHb | d35 | 2.26692328 |
| TmHb | d35 | 7.78628764 |
| Nv | Nv | 0 |
| Nv | Nv | 0.17275528 |
| Nv | Nv | 0 |
| Nv | Nv | 0.11840906 |
| Nv | Nv | 0 |

**Supplementary Table 7**

**Raw data for Figure 4**

| **Infection** | **Condition** | **Tm Burden** | **Hb Burden** | ***Retnlb* relative expression** | **Goblet cells average/crypt** |
| --- | --- | --- | --- | --- | --- |
| Tm | PBS_Alum | 122 | 0 | 3.90611946 |  |
| Tm | PBS_Alum | 87 | 0 | 4.8918101 | 9 |
| Tm | PBS_Alum | 115 | 0 | 0.94009917 | 9.8 |
| Tm | PBS_Alum | 103 | 0 | 0.41020188 | 7.2 |
| Tm | PBS_Alum | 95 | 0 | 0.59447007 | 3.6 |
| Tm | ES_Alum | 0 | 0 | 5.45795839 | 11.5 |
| Tm | ES_Alum | 0 | 0 | 3.37391609 | 13.1 |
| Tm | ES_Alum | 0 | 0 | 4.26981085 | 15.4 |
| Tm | ES_Alum | 0 | 0 | 2.10578384 | 18.1 |
| Tm | ES_Alum | 0 | 0 | 2.45431893 | 11.7 |
| TmHb | PBS_Alum | 89 | 55 | 0.45640684 | 5.3 |
| TmHb | PBS_Alum | 99 | 71 | 2.62351224 | 14.5 |
| TmHb | PBS_Alum | 77 | 46 | 0.30205991 | 5.7 |
| TmHb | PBS_Alum | 120 | 35 | 0.30473566 | 4.5 |
| TmHb | PBS_Alum | 121 | 50 | 0.88164653 | 5.5 |
| TmHb | ES_Alum | 0 | 41 | 2.23795678 | 10.3 |
| TmHb | ES_Alum | 0 | 38 | 2.04779183 | 11.3 |
| TmHb | ES_Alum | 0 | 43 | 3.50691069 | 10.5 |
| TmHb | ES_Alum | 0 | 76 | 2.5241127 | 9.3 |
| TmHb | ES_Alum | 0 | 72 | 0.19061995 | 11.5 |

**Supplementary Table 8**

**Raw data for Figure 5A. Worm burdens (N^o^ Tm worms/mouse)**

| **Group** | **Tm worm count** |
| --- | --- |
| Tm | 58 |
| Tm | 111 |
| Tm | 38 |
| Tm | 154 |
| TmHb | 92 |
| TmHb | 109 |
| TmHb | 84 |
| TmHb | 142 |

**Supplementary Table 9**

**Raw data for Figure 5C. Worm lengths in cm**

| **Group** | **Sex** | **Length (cm)** |
| --- | --- | --- |
| Tm | M | 1.567 |
| Tm | M | 1.525 |
| Tm | M | 1.675 |
| Tm | M | 1.318 |
| Tm | M | 1.798 |
| Tm | M | 1.837 |
| Tm | M | 1.82 |
| Tm | M | 1.72 |
| Tm | M | 1.426 |
| Tm | M | 1.487 |
| Tm | M | 1.861 |
| Tm | M | 1.623 |
| Tm | M | 1.926 |
| Tm | M | 1.868 |
| Tm | M | 1.734 |
| Tm | M | 1.922 |
| Tm | M | 1.659 |
| Tm | F | 1.584 |
| Tm | F | 1.203 |
| Tm | F | 1.67 |
| Tm | F | 2.058 |
| Tm | F | 1.87 |
| Tm | F | 1.866 |
| Tm | F | 1.837 |
| Tm | F | 1.956 |
| Tm | F | 1.96 |
| Tm | F | 1.889 |
| Tm | F | 1.429 |
| Tm | F | 1.487 |
| Tm | F | 1.77 |
| Tm | F | 2.045 |
| Tm | F | 1.865 |
| Tm | F | 1.769 |
| Tm | F | 1.79 |
| TmHb | M | 1.545 |
| TmHb | M | 1.786 |
| TmHb | M | 1.882 |
| TmHb | M | 1.752 |
| TmHb | M | 1.778 |
| TmHb | M | 1.73 |
| TmHb | M | 1.447 |
| TmHb | M | 1.826 |
| TmHb | M | 1.9 |
| TmHb | M | 1.56 |
| TmHb | M | 1.663 |
| TmHb | M | 2.072 |
| TmHb | M | 1.567 |
| TmHb | M | 1.555 |
| TmHb | M | 2.163 |
| TmHb | M | 1.958 |
| TmHb | M | 1.953 |
| TmHb | M | 1.472 |
| TmHb | F | 1.887 |
| TmHb | F | 2.211 |
| TmHb | F | 2.049 |
| TmHb | F | 1.676 |
| TmHb | F | 1.719 |
| TmHb | F | 1.934 |
| TmHb | F | 2.085 |
| TmHb | F | 1.807 |
| TmHb | F | 2.4 |
| TmHb | F | 1.779 |
| TmHb | F | 2.104 |
| TmHb | F | 1.972 |
| TmHb | F | 1.879 |
| TmHb | F | 1.74 |
| TmHb | F | 1.977 |
| TmHb | F | 2.3 |
| TmHb | F | 1.797 |

**Supplementary Table10**

**Raw data for Figure 5D. Percent of fecund female worms/mouse**

| **Group** | **Percent Fecund (%)** |
| --- | --- |
| Tm | 0 |
| Tm | 20 |
| Tm | 40 |
| Tm | 80 |
| TmHb | 80 |
| TmHb | 100 |
| TmHb | 0 |
| TmHb | 40 |

**Supplementary Table 11**

**Raw data for Figure 5E.**

| **Group** | **Egg Count** |
| --- | --- |
| Tm | 1.65 |
| Tm | 72.5 |
| Tm | 122.5 |
| Tm | 35 |
| Tm | 2.5 |
| Tm | 7.5 |
| Tm | 15 |
| TmHb | 125 |
| TmHb | 125 |
| TmHb | 5 |
| TmHb | 15 |
| TmHb | 135 |
| TmHb | 12.5 |
| TmHb | 31.7 |
| TmHb | 48.3 |
| TmHb | 33.3 |
| TmHb | 66.7 |
| TmHb | 15 |
